# Correlation between CO_2_ storage at the last minute of gas insufflation and area of retroperitoneal lacuna during retroperitoneal laparoscopic radical nephrectomy

**DOI:** 10.1186/s12871-016-0208-z

**Published:** 2016-07-22

**Authors:** Jian-jun Hu, Ya-Hua Liu, Chan-juan Yu, Nuerbolati Jialielihan

**Affiliations:** Anesthesia Department of Xinjiang Medical University Affiliated Tumor Hospital, No. 789, Suzhoudong Road, Urumqi, 830011 Xinjiang China

**Keywords:** Retroperitoneal laparoscopic radical nephrectomy, CO_2_ absorption, Retroperitoneal lacuna, Correlation

## Abstract

**Background:**

Adequate operation interspace is the premise of laparoscopy, and carbon dioxide (CO_2_) was an ideal gas for forming lacuna. A retroperitoneal space is used to form operation interspace in retroperitoneal laparoscopic radical nephrectomy by making ballooning, and the retroperitoneal space has no relative complete and airtight serous membrane, therefore CO_2_ absorption may be greater in retroperitoneal than transperitoneal laparoscopic radical nephrectomy. Excess CO_2_ absorption may induce hypercapnemia and further cause physiopathological change of respiratory and circulatory system. Therefore, exact evaluation of amount of CO_2_ which is eliminated from body via minute ventilation is important during retroperitoneal laparoscopic radical nephrectomy. The aim of the paper is to study the correlation between CO2 storage at the last minute of gas insufflation and area of retroperitoneal lacuna during retroperitoneal laparoscopic radical nephrectomy.

**Methods:**

Forty ASA I/II patients undergoing retroperitoneal laparoscopic radical nephrectomy were enrolled. CO_2_ storage at the last minute of gas insufflation and area of a retroperitoneal lacuna were observed. Linear correlation and regression were performed to determine the correlation between them.

**Results:**

There was positive correlation between CO_2_ storage at the last minute of gas insufflation and area of retroperitoneal lacuna (r = 0.880, *P* = 0.000), and the equation of linear regression was y = −83.097 + 0.925x (R^2^ = 0.780, *t* = 11.610, *P* = 0.000).

**Conclusions:**

Amount of CO_2_ which is eliminated from body via mechanical ventilation could be calculated by measuring the area of retroperitoneal lacuna during retroperitoneal laparoscopic radical nephrectomy, and an anesthetist should be aware of the size of lacuna to predict high CO_2_ storage at the last minute of gas insufflation.

## Background

Laparoscopic radical nephrectomy has gradually replaced conventional open nephrectomy with the improvement of operation technique and laparoscopic apparatus [1–3]. Adequate operation interspace is the premise of laparoscopy, and carbon dioxide (CO_2_) was an ideal gas for forming lacuna [4]. A retroperitoneal space is used to form operation interspace in retroperitoneal laparoscopic radical nephrectomy by making ballooning, and the retroperitoneal space has no relative complete and airtight serous membrane [5], therefore CO_2_ absorption may be greater in retroperitoneal than transperitoneal laparoscopic radical nephrectomy [6]. Excess CO_2_ absorption may induce hypercapnemia [7] and further cause physiopathological change of respiratory and circulatory system [8, 9]. This is harmful for patients with renal cancer because they are generally complicated with the dysfunction of critical organs. In the paper, the correlation between CO_2_ storage at the last minute of gas insufflation and the area of retroperitoneal lacuna in retroperitoneal laparoscopic radical nephrectomy was studied, and the aim was to evaluate exactly amount of CO_2_ which is eliminated from body via minute ventilation and further provide useful information for intraoperative management and mechanical ventilation strategy.

## Methods

### Participants

Forty ASA I/II patients between 22 and 70 years old undergoing retroperitoneal laparoscopic radical nephrectomy were enrolled in the study. All patients were treated by the same group of surgeons. Among them, 25 patients were male and 15 patients were female, and the body mass index (BMI) was 22.19 ± 0.47.

### Anesthesia method

All the patients were not administered with medicine and their respiratory and circulatory functions were evaluated before operation. They received routine general anesthesia, and Electrocardiograph (ECG), blood oxygen saturation (SPO_2_) and blood pressure (BP) were monitored. Three arterial blood gas (ABG) samples were collected at the last minute of gas insulation, and partial pressure of carbon dioxide in artery (PaCO_2_) was then measured. Meanwhile, end-tidal partial pressure of carbon dioxide (PetCO_2_) was monitored at the corresponding time point. The mean arterial pressure and heart rate were measured before anesthesia induction, after anesthesia induction, at the time point when retroperitoneal lacuna was successfully formed, and at the end of operation.

Anesthesia was induced with propofol (2 mg/kg), fentanyl (4 ug/kg), and cisatracurium (0.2 mg/kg). After oral trachea cannula, patients underwent mechanical ventilation with a tidal volume of 8 ml/kg. PetCO_2_ was maintained at ≤50 mmHg by regulating respiratory frequency. Anesthesia was maintained by the method of intravenous-inhalation combined anesthesia with propofol (4–6 mg/kg∙h), sevoflurane (1.5–3 %), fentanyl (2 μg /kg∙h) and cisatracurium (0.1–0.15 mg/kg∙h).

### Process of retroperitoneal lacuna formation

Jackknife position was adopted to protrude operation site after anesthesia. A retroperitoneal lacuna was formed by making ballooning with CO_2_ at an insufflation pressure of 12–13 mmHg for 30 min, and retroperitoneal tissues were separated gradually to get a regular triaxial ellipsoid. The surgeons were asked to try their best to make the surface of lacuna smooth.

### Computing method

#### Computing the area of retroperitoneal lacuna

**Fig. 1:**
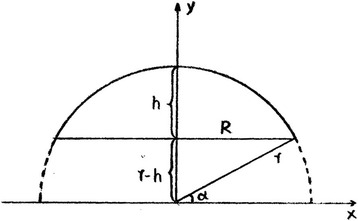
Computation of the area of retroperitoneal lacuna. “R” refers to long diameter of balloon, “r” refers to wide diameter, and “h” refers to high diameter. “X” refers to X axis, and “y” refers to y axis

The area was generally stable after retroperitoneal lacuna was formed. The surface area of sphere was minimal at the condition of same volume, which could make the absorption of CO_2_ minimal. The surgeons were asked to try their best to make the surface of lacuna smooth. Therefore, the lacuna could be regarded as a triaxial ellipsoid. The surface area of lacuna could be calculated by the method of calculus after measuring the radius, which was equal to the area of CO_2_ absorption. The three radii of triaxial ellipsoid were simultaneously measured when designing operating field. The final formula was S = π (R^2^ + h^2^) for computing the area of retroperitoneal lacuna (Fig. 1).

#### Computing CO_2_ storage at the last minute of gas insufflation during retroperitoneal laparoscopic radical nephrectomy

CO_2_ storage (VCO_2_) at the last minute of gas insufflation was able to be calculated using the below equation.

$$ \begin{array}{cc}\hfill {\mathrm{VCO}}_2={\mathrm{k}}_2\cdotp \mathrm{V}\mathrm{A}\cdotp {\mathrm{PaCO}}_2,\hfill & \hfill \mathrm{V}\mathrm{A}=\mathrm{V}\mathrm{T}-\mathrm{V}\mathrm{D}\hfill \end{array} $$

Where PaCO_2_ is arterial partial pressure of carbon dioxide in kpa; VT is expired tidal volume in ml; VD is physiologic dead space in percent of tidal volume. K_2_ is the constant for arterial partial pressure of carbon dioxide transformed into concentration of carbon dioxide. The value of k_2_ is 8.16 when VA is expressed in ml and PaCO_2_ in kpa, on the condition of standard atmosphere pressure, 37 °C of body temperature and saturation of water vapor [10]. Physiologic dead space in percent of tidal volume (VD) is calculated in the following formula.

$$ \mathrm{V}\mathrm{D}=\mathrm{V}\mathrm{T}\times \left({\mathrm{PaCO}}_2-{\mathrm{PetCO}}_2\right)/{\mathrm{PaCO}}_2. $$

Where PaCO_2_ is arterial partial pressure of carbon dioxide in kpa and PetCO_2_ is end tidal partial pressure of carbon dioxide in kpa.

PaCO_2_ and PetCO_2_ were observed at the last minute of gas insufflation. Correspondently, VCO_2_ was computed at the time point in ml.

### Statistical analysis

The statistical analyses were carried out with the SPSS version 17.0 for Windows (SPSS Inc., USA). All the variables were expressed as mean ± SD. Linear correlation and regression were performed to determine the correlation between amount of CO_2_ which is eliminated from body via mechanical ventilation and the area of retroperitoneal lacuna and obtain the equation of linear regression. Significance was set at *P* < 0.05.

## Results

Retroperitoneal laparoscopic radical nephrectomy was successfully performed in all patients. The operation time ranged from 148 to 152 min with a mean of 150.12 ± 1.86, and was almost same for all patients. Subcutaneous emphysema did not occur. In addition, CO_2_ insufflation time and insufflation pressure were constant in the study. Therefore, the effects of operation time, subcutaneous emphysema, insufflation time and insufflation pressure on CO_2_ absorption were not studied in the paper. The mean arterial pressure and heart rate were not significantly different between different time points (all *P* > 0.05, Table 1). The results indicated that the conditions of patients were stable during the whole operation.

**Table 1:** Mean arterial pressure and heart rate at different time points

	Mean arterial pressure (mmHg)	Heart rate
Before anesthesia induction	67 ± 5.8	79 ± 11.2
After anesthesia induction	61 ± 4.9	73 ± 11.2
Time point when retroperitoneal lacuna was successfully formed	64 ± 5.2	72 ± 11.2
End of operation	63 ± 5.1	76 ± 11.2

CO_2_ storage at the last minute of gas insufflation and the area of retroperitoneal lacuna were shown in Table 2. The mean of amount of CO_2_ which is eliminated from body via mechanical ventilation was 479.15 ± 46.71 ml for the 40 patients, the mean of the area of retroperitoneal lacuna was 686.52 ± 21.16 cm^2^, the mean of VT was 453.25 ± 97.91 ml, the mean of PaCO_2_ was 5.54 ± 0.22 kpa, and the mean of PetCO_2_ was 4.45 ± 0.35 kpa. The result of linear correlation showed that there was positive correlation between amount of CO_2_ which is eliminated from body via minute ventilation and area of retroperitoneal lacuna (r = 0.880, *P* = 0.000), and the equation of linear regression was y = −83.097 + 0.925x (R^2^ = 0.780, *t* = 11.610, *P* = 0.000).

**Table 2:** CO_2_ storage (VCO_2_) at the last minute of gas insulation and the area of retroperitoneal lacuna (S)

	VCO_2_ (ml)	S (cm^2^)	PaCO_2_ (kpa)	PetCO_2_ (kpa)	VT (ml)		VCO_2_ (ml)	S (cm^2^)	PaCO_2_ (kpa)	PetCO_2_ (kpa)	VT (ml)
1	499.39	767.7	5.60	4.67	440	21	495.53	765.9	5.60	4.40	550
2	642.61	1125.7	6.00	5.07	340	22	421.45	671.6	5.47	4.27	380
3	855.45	1374.8	6.40	5.33	330	23	510.43	731.2	5.47	4.27	600
4	380.65	702.9	5.47	4.80	480	24	392.63	613.4	5.33	4.27	500
5	458.76	734.4	5.60	4.40	370	25	389.61	575.6	5.33	4.13	700
6	479.13	702.8	5.47	4.27	470	26	375.36	526.4	5.20	3.87	500
7	774.63	1010.7	5.87	4.67	390	27	538.35	734.9	5.47	4.27	680
8	325.96	556.5	5.33	4.27	450	28	396.27	563.6	5.20	3.87	400
9	462.64	734.5	5.47	4.40	450	29	356.38	580.6	5.33	4.13	550
10	482.75	765.8	5.60	4.40	420	30	524.46	641.1	5.47	4.27	360
11	502.24	832.8	5.73	4.53	540	31	393.39	501.2	5.20	4.00	340
12	495.54	765.9	5.47	4.27	420	32	418.39	669.2	5.47	4.27	650
13	500.19	732.7	5.47	4.27	380	33	459.52	642.4	5.47	4.27	350
14	484.52	764.2	5.60	4.40	320	34	518.18	834.4	5.73	4.67	380
15	450.26	702.9	5.47	4.27	450	35	536.68	902.8	5.87	5.07	450
16	526.72	867.8	5.73	4.67	430	36	385.63	765.9	5.60	5.33	400
17	489.39	641.9	5.47	4.27	350	37	374.9	700.1	5.47	5.20	380
18	472.94	698.6	5.47	4.27	480	38	558.38	799.2	5.73	4.53	660
19	400.75	701.1	5.47	4.27	390	39	400.12	641.4	5.47	4.40	450
20	530.23	734.9	5.60	4.40	450	40	505.57	765.9	5.60	4.67	500

Notes: *VCO*_*2*_ CO_2_ storage, *S* Area of retroperitoneal lacuna, *PaCO*_*2*_ Partial pressure of carbon dioxide in artery, *PetCO*_*2*_ End-tidal partial pressure of carbon dioxide, *VT* Expired tidal volume

## Discussion

Retroperitoneal laparoscopic radical nephrectomy has special pathophysiologic change compared with transperitoneal laparoscopic radical nephrectomy. Firstly, retroperitoneum is a potential lacouna and has no relative complete and airtight serous membrane. Secondly, subcutaneous and connective tissue are separated during the course of establishing retroperitoneum, which makes subcutaneous capillaries torn and CO_2_ is then absorbed and solved easily in blood. Thirdly, retroperitoneal laparoscopic radical nephrectomy adopts Jackknife position, which can reduce the elimination of CO_2_. All these lead to greater CO_2_ absorption in retroperitoneal than transperitoneal laparoscopic radical nephrectomy. Therefore, an anesthetist should be aware of the size of lacuna to predict high amount of CO_2_ which is eliminated from body via mechanical ventilation.

In conclusion, we analyzed the correlation between amount of CO_2_ which is eliminated from body via minute ventilation and area of retroperitoneal lacuna and obtain the equation of linear regression in the paper. The result was instructive for preoperative evaluation of CO_2_ storage at the last minute of gas insufflation and was then helpful in determining whether open nephrectomy should be adopted or not and evaluating safety of patients.

## Conclusions

Amount of CO_2_ which is eliminated from body via mechanical ventilation could be calculated by measuring the area of retroperitoneal lacuna in retroperitoneal laparoscopic radical nephrectomy, and an anesthetist should be aware of the size of lacuna to predict high CO_2_ storage at the last minute of gas insufflation.

## Abbreviations

CO_2_, carbon dioxide; BMI, body mass index; ECG, electrocardiograph; SPO_2_, blood oxygen saturation; PaCO_2_, partial pressure of carbon dioxide in artery; PetCO_2_, end-tidal partial pressure of carbon dioxide; VCO_2_, CO_2_ storage; BP, blood pressure; ABG, arterial blood gas.
